# Atomistic Study of Lipid Membranes Containing Chloroform: Looking for a Lipid-Mediated Mechanism of Anesthesia

**DOI:** 10.1371/journal.pone.0052631

**Published:** 2013-01-02

**Authors:** Ramon Reigada

**Affiliations:** Departament de Química Física and Institut de Química Teòrica i Computacional (IQTCUB), Universitat de Barcelona, Barcelona, Spain; Emory University School of Medicine, United States of America

## Abstract

The molecular mechanism of general anesthesia is still a controversial issue. Direct effect by linking of anesthetics to proteins and indirect action on the lipid membrane properties are the two hypotheses in conflict. Atomistic simulations of different lipid membranes subjected to the effect of small volatile organohalogen compounds are used to explore plausible lipid-mediated mechanisms. Simulations of homogeneous membranes reveal that electrostatic potential and lateral pressure transversal profiles are affected differently by chloroform (anesthetic) and carbon tetrachloride (non-anesthetic). Simulations of structured membranes that combine ordered and disordered regions show that chloroform molecules accumulate preferentially in highly disordered lipid domains, suggesting that the combination of both lateral and transversal partitioning of chloroform in the cell membrane could be responsible of its anesthetic action.

## Introduction

The effect of chloroform (CHCL_3_) on lipid bilayers has been extensively studied during the past decades, in part motivated by the desire to understand its toxicity in cells [1]. The damaging effect of chloroform has been attributed to its ability to modify the properties of the cell membrane lipid matrix. Intriguingly, experiments with model bilayers have shown that the influence of chloroform is modulated by the bilayer composition [2], [3]. More specifically, Regen et al. have recently reported that chloroform loosens cholesterol-rich lipid membranes whereas it has the opposite effect in cholesterol-poor bilayers [4], [5]. Molecular Dynamic (MD) simulations have unveiled the molecular mechanism that explains this differential behavior: due to its particular interaction with cholesterol, chloroform induces a strong chain disordering in liquid-ordered (*lo*) membrane phases containing cholesterol, whereas it promotes chain ordering in liquid-disordered (*ld*) membrane phases with low cholesterol contents [6].

The fact that chloroform has a potent anesthetic effect is also an important motivation to study its influence on lipid bilayers. Although there is a general consensus that anesthetic drugs modulate the activity of ion channels involved in the neurotransmission system, the molecular mechanism underlying this modulation has been a controversial issue [7]. Two different theories have been historically debated. One hypothesis is in favor of direct interactions (binding) of the anesthetic compounds with specific protein receptor sites [8], [9]. Actually, protein receptors have been found for many anesthetics. However, the diversity of molecular structures of anesthetic compounds and the wide variety of responding systems are difficult to reconcile with the specific binding concept and suggested an alternative hypothesis based on a nonespecific mechanism through an alteration of the global physical properties of the membrane lipid matrix (thickness, area, chain ordering, melting temperature) [10]–[13]. This hypothesis is also supported by the Meyer-Overton's rule that correlates the potency of anesthetic drugs with how well they dissolve in olive oil. The main drawback of the lipid-mediated mechanisms is that nearly all the experiments that analyze the effects of anesthetic compounds on model lipid bilayers have been made at anesthetic concentrations much higher than those clinically required to promote block nerve conduction. Instead, at pharmacological concentrations, little effects on the global properties of synthetic membranes are observed [14].

Despite these considerations, it seems that the molecular mechanism of anesthesia was controversial only from simplistic standpoints. Recent progress in the understanding of biomembranes indicates that the origin of the anesthetic action is probably complex and involves a combination of the two confronted views. For example, R. Cantor [15], [16] demonstrated that an alteration of the internal lateral pressures of the lipid membrane may strongly modify the work done by a protein to open or close its ion channel. This suggests a very simple, yet powerful idea: if an anesthetic compound inserts into the membrane it will change its lateral pressure profile, so that the opening/closing dynamics of the ion channels involved in the neurotransmission process will be modified. Thus, the effect of the anesthetic molecules on the proteins responsible for nerve conduction is not caused by the modification of the global physical properties of the membrane but indirectly performed by the alteration of local mechanical properties of the lipid matrix. Analogously, alterations of the transmembrane electrostatic potential could be also responsible for the alteration of protein function. Additionally, the anesthetic action is probably performed at two levels: at the local scale, the lipid environment of a given protein significantly affects its functionality, and at the global scale, lipid organization affects sorting and activation of membrane proteins. The anesthesic action may also involve these two scales.

Molecular Dynamics has been recently used to investigate the effect of anesthetics like chloroform [6], halothane [17], [18], or ketamine [19], on simple lipid membranes. Although limited to short time (≈200 ns) and length (≈10 nm) scales, MD simulations provide a description of the resulting membrane alterations, and more importantly, the responsible mechanisms of these changes can be unveiled at the molecular level. One important feature is that anesthetic molecules use to partition into different sections of the bilayer, thus modifying its transversal properties. The computation of these properties constitutes an additional advantage of MD since most of them are not experimentally accessible. As a remarkable example, membrane lateral pressure profiles have been shown to be significantly modified by ketamine [19]. It is important to notice here the relevant contribution by Ollila et al. [20] on the calculation of the internal pressure field from MD simulations of membrane systems displaying curvature, lipid heterogeneity, or the presence of inserted proteins. All these scenarios are of interest in the context of the anesthetic issue.

In this paper, a collection of systematic and extensive MD simulations is presented with a twofold aim: the characterization of the effects of chloroform in different lipid phases, and the exploration of plausible molecular mechanisms to explain its anesthetic action. To do so, a triple set of simulations at different chloroform fractions has been performed for homogeneous ordered and homogeneous disordered bilayers, and for structured membranes combining regions of ordered and disordered lipid phases. In order to discriminate possible causes of the anesthetic action, most of the simulations have been duplicated using carbon tetrachloride (CCl_4_), a non-anesthetic molecule with a similar structure than chloroform. In the homogeneous bilayers, special attention is paid to the transversal properties, mainly to lateral pressure and electrostatic potential profiles. Interestingly, chloroform and carbon tetrachloride do not partition likewise inside the membrane and cause different effects on its transversal properties. Additionally, simulations of structured membranes show that chloroform displays a clear preference to accumulate in disordered lipid phases. The combination of these two observations indicates a plausible lipid-mediated mechanism of anesthesia by chloroform.

## Methods

### Simulations of Homogeneous Model Membranes

Atomic-scale molecular dynamics (MD) simulations were carried out for two different types of phosphatidylcholine (PC) membrane systems. Pure DOPC (PC with two unsaturated 18∶1 oleoyl chains) bilayers, representative of a *ld* phase, and bilayers of DSPC molecules (PC with two saturated 18∶0 stearyl chains) mixed with 20%mol of cholesterol (DSPC/Chol) that represent a *lo* phase. All lipid bilayers were composed of a total of 128 PC molecules together with 32 Chol molecules in the case of DSPC/Chol bilayers, and were sufficiently hydrated with 6186 water molecules. Each bilayer system was run with 12, 32, 64, 256 and 512 chloroform (chlf) molecules, or 12, 32, 64 and 512 carbon tetrachloride (ctcl) molecules. Organohalogen molecules were included in the equilibrated membrane systems in three different manners: in the middle of the two leaflets, in the water/membrane interface and in the aqueous bulk phase. In the three cases, organohalogen molecules were finally absorbed by the bilayer and redistributed inside it after a few nanoseconds.

The simulations were performed using the GROMACS v.3.3.1 software package [21]. I used the standard united-atom force-field parameters by Berger et al. [22] for PC molecules, together with the adaptation performed by Bachar et al. [23] for the double-bond region of DOPC (see [24] for details), the description of Holtje et al. [25] for cholesterol, and for chlf and ctcl the force field parameters were adapted from GROMOS96 [26] (see [6] for more details). The Simple Point Charge (SPC) model [27] was employed for water. SETTLE and LINCS algorithms were used to preserve bond lengths in water and lipid molecules, respectively. A single 1.0 nm cutoff distance was used for Lennard-Jones interactions. The long-range electrostatic interactions were handled using the particle-mesh Ewald method [28]. Periodic boundary conditions were used in all three directions, and the integration step was set to 2 fs.

The simulations were carried out in the NpT ensemble at T = 310 K and an anisotropic p = 1 atm. All simulated membranes are in a fluid state. Temperature and pressure were controlled by using the weak coupling method [29] with relaxation times set to 0.6 and 1.0 ps, respectively. Equilibration of each bilayer system was determined by monitoring membrane area and chlf (or ctcl) density profiles, and in all cases it was achieved before 20 ns. Additional 130 ns were run and used for analysis. This simulation protocol has been successfully applied in previous MD simulations [30], [31], and the obtained values for structural membrane properties such as area per molecule, membrane thickness, and scattering form factors are well in line with experimental data (see [30] for details).

### Membrane Transversal Properties

The local pressure in a system consisting of interacting particles is defined using the local stress tensor. The lateral pressure profile, P_L_(z) is computed as the lateral components of the pressure tensor, (P_xx_+P_yy_)/2, at different z values. Technically, P_L_(z) profiles were evaluated by re-running the simulation trajectories with a Gromacs 4.0.2 version and applying the Irving-Kirkwood contour. SHAKE constraints were used and electrostatic interactions were truncated at 2 nm. This protocol has been successfully used in other works and it is detailed in [32]. Cubic splines are used to smooth the obtained profiles.

The membrane electric potential profile, V(z), is computed for each membrane system as the double integral of its charge density distribution ρ_q_(z); namely by integrating twice the Poisson equation,
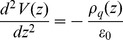
where ε_0_ is the vacuum permittivity. Following the procedure detailed in [33], the potential and electric field are chosen to be zero at the center of mass of the bilayer (z = 0) as boundary conditions for the integration. Since the position of the bilayer center of mass fluctuates during the simulation, the positions of all atoms in the system are centered with respect to it in each analyzed system configuration.

The potential of mean force (PMF) for the organohalogen compounds inside the bilayer is estimated from the corresponding density profiles, ρ(z), using the standard expression 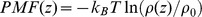
_,_ where ρ_0_ is the mass density in the reference state, here taken as the center of the bilayer. This method is reliable for molecules that significantly penetrate into the membrane, as it is clearly the case for chlf and ctcl.

All the profiles are computed by dividing the system in 0.1 nm thick slices along the z-axis, and are plotted in a scaled distance with respect to the bilayer center, z_esc_, where the density maxima of phosphate groups are fixed at z_esc_ = ±1. It is important to notice that pressures and electric potential profiles require production runs as long as 100 ns to achieve a correct convergence [33]. Here, trajectories of 130 ns have been used.

### Structured Model Membranes

Some simulations were run for a structured bilayer consisting of a *lo* nanodomain surrounded by a *ld* phase. This system was generated as follows: an equilibrated DSPC/Chol bilayer was replicated 2×2 and all Chol and DSPC molecules that were further than a given distance from the center of the (x,y) simulation plane were removed or changed to DOPC, respectively. The mentioned distance was chosen so that the final system contained 256 DSPC, 256 DOPC and 64 Chol molecules, evenly distributed in the two leaflets. As a result, a rather circular patch of DSPC/20%Chol surrounded by DOPC was obtained.

Following a similar procedure, a second heterogeneous system is generated, consisting in a small *ld* patch of 64 DOPC molecules surrounded by a *lo* phase of 448 DSPC and 112 Chol molecules. Both heterogeneous membrane systems are also generated and simulated containing 128 chlf molecules (equivalent to the homogeneous systems with 32 chlf). Simulations for the *lo* and *ld* nanodomains were run for 200 and 100 ns, respectively. In both cases, the same simulation protocol than in homogeneous bilayers was applied.

### Voronoi Tessellation

**Figure 1:**
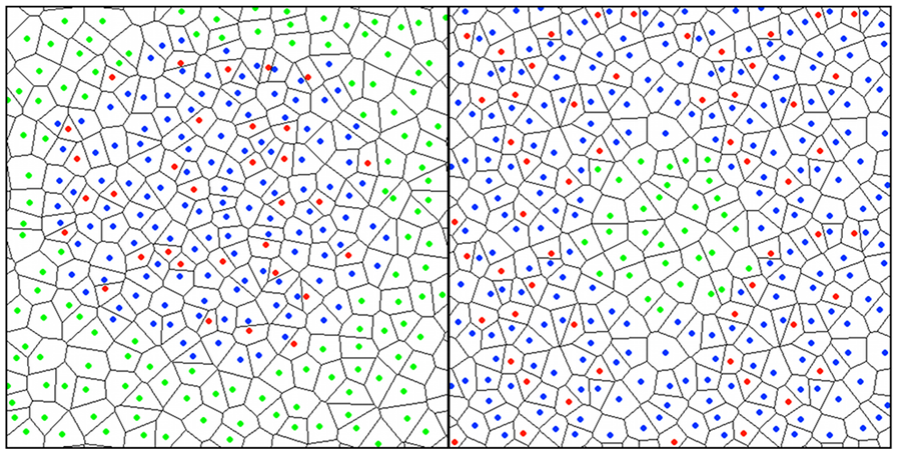
Voronoi tessellation of a leaflet of the lo (left) and small ld (right) nanodomain membranes after 20 ns of simulation. Key atoms for each molecule are plotted: DOPC (green), DSPC (blue) and Chol (red).

Structural analysis of multicomponent membranes is performed by the use of Voronoi tessellation. To do so, key atoms of the PC and Chol molecules of a given leaflet are projected onto a plane. The carbon group linking the glycerol and the phosphate groups is chosen for the PC molecules whereas the hydroxyl group represents Chol molecules. The Voronoi tessellation is performed for each leaflet according to the mentioned projections (see [34] for details). Examples of tessellated membrane leaflets corresponding to the two kind of heterogeneous bilayers simulated in this paper are shown in Figure 1.

Since each Voronoi polygon is associated with an individual molecule, membrane properties like area per molecule, membrane thickness, or acyl chain ordering can be computed and differential values can be assigned to each component of the membrane. In particular, membrane thickness is computed for each PC molecule in the bilayer as the distance between its phosphorous group and the phosphorous group of the transbilayer PC neighbor molecule (additional details in [33]).

## Results

### Structural Changes in Homogeneous Model Membranes

Some structural parameters of the simulated homogeneous membranes are summarized in Table 1. Internal ordering of lipid membranes is characterized by the deuterium order parameter, S_CD_, defined for each Carbon group of the two lipid tails [35]. Its average for all Carbon groups involved in single bonds, <-S_CD_>, quantifies the global lipid tail ordering. Adding chlf decreases lipid ordering for a DSPC/Chol bilayer, and increases ordering for a DOPC membrane (Table 1). Since DOPC chains become more ordered the inclusion of chlf increases the thickness of DOPC bilayers. Instead, in a DSPC/Chol bilayer, chlf disorders lipid molecules, so that the membrane becomes thinner (see Table 1). In both cases, chlf molecules add some extra volume to the membrane system and this results in larger values for the area per molecule (Table 1). These observations show that chloroform affects in a different manner DOPC and DSPC/Chol bilayers, namely, *ld* and *lo* lipid phases. Such differential behavior is due to the unlike interaction of chlf with PC and Chol. Chlf molecules can be easily adapted to the flexible PC acyl chains whereas they cannot be laterally accommodated close to rigid cholesterol molecules. This conclusion was already reported in [6] for saturation conditions and it is here corroborated for lower chlf concentrations.

**Table 1:** Average values of structural properties of the simulated homogeneous membranes^a^^,^ b.

	A/PC (nm^2^±0.005)	A/Chol (nm^2^±0.005)	Thickness (nm±0.02)	<-S_CD_> (±0.003)	P_1_ (k_B_T/nm±1)	P_2_ (k_B_T±2)
Pure bilayers	0.701	-	3.79	0.108	−6.2	−15.4
	**0.452**	**0.363**	**5.02**	**0.341**	**−4.2**	**−6.6**
12 chlf	0.714	-	3.87	0.108	−6.2	−15.7
	**0.454**	**0.358**	**5.06**	**0.340**	**−5.0**	**−9.6**
12 ctcl	0.703	-	3.85	0.109	−6.2	−15.6
	**0.454**	**0.350**	**5.08**	**0.340**	**−7.1**	**−11.0**
32 chlf	0.726	-	3.75	0.112	−6.3	−15.5
	**0.473**	**0.368**	**4.99**	**0.324**	**−8.0**	**−17.8**
32 ctcl	0.717	-	3.71	0.119	−6.3	−14.9
	**0.502**	**0.389**	**4.78**	**0.330**	**−7.0**	**−16.9**
64 chlf	0.739	-	3.77	0.118	−5.8	−14.9
	**0.496**	**0.392**	**4.87**	**0.306**	**−8.0**	**−20.6**
64 ctcl	0.739	-	3.78	0.120	−6.7	−16.0
	**0.510**	**0.410**	**4.71**	**0.312**	**−8.7**	**−22.2**
256 chlf	0.803	-	3.81	0.128	−5.3	−14.6
	**0.626**	**0.434**	**4.57**	**0.253**	**−8.6**	**−22.1**
512 chlf	0.912	-	3.91	0.132	−5.2	−14.8
	**0.754**	**0.457**	**4.50**	**0.218**	**−8.5**	**−22.7**
512 ctcl	0.947	-	4.08	0.133	−8.4	−20.9
	**0.785**	**0.478**	**4.64**	**0.220**	**−12.2**	**−32.8**

a Values in normal characters stand for DOPC bilayers, whereas results for DSPC/Chol systems are shown in bold.

b Errors are estimated as twice the standard deviation. The maximum error bar for each property is given in the table.

Regarding the relevance of these findings on the chlf anesthetic action, two main drawbacks have to be noticed. First, membrane structural modifications are evident at saturation conditions, but almost insignificant at clinically relevant concentrations: membranes with 12 chlf molecules show negligible variations in area per molecule, thickness and lipid ordering. Small but discernible (about 3–5%) changes of some properties can be found when adding 32 chlf molecules (already above clinical concentrations). However, the magnitude of these changes cannot be biologically relevant since they can be accomplished by temperature fluctuations. Another important drawback corresponds to the results obtained with carbon tetrachloride. All physical membrane properties are similarly modified when adding ctch molecules, so the anesthetic action of chlf cannot be explained by the alteration of these parameters. Other membrane properties like membrane fluidity, in-plane ordering, and lipid tail and headgroup orientations have been analyzed with similar results (data not shown).

### Lateral Pressure and Electrostatic Potential Profiles

**Figure 2:**
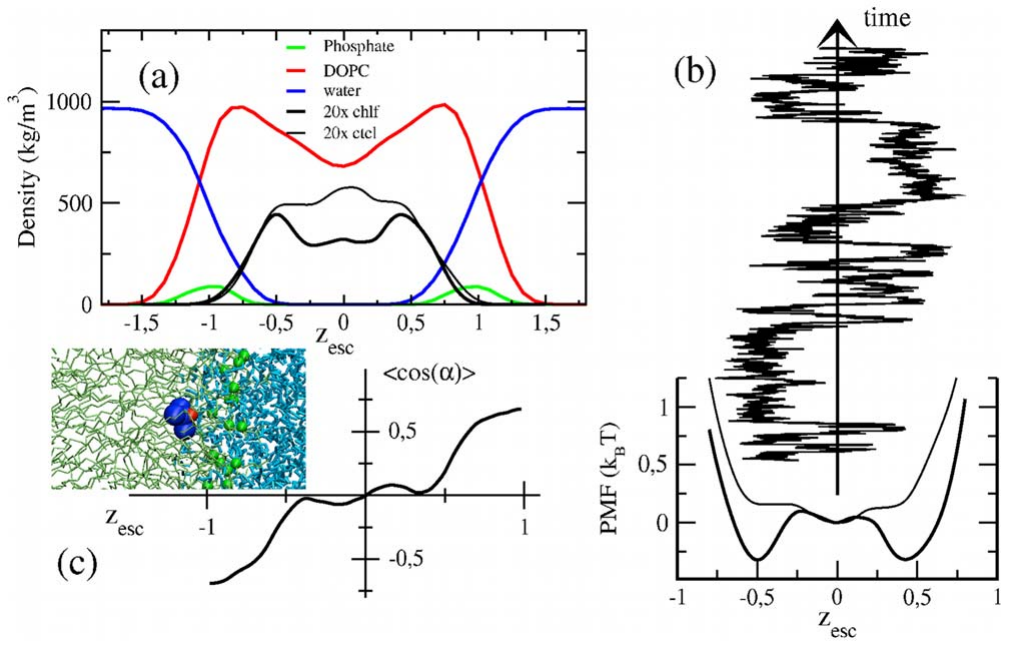
Distribution of organohalogen molecules inside a bilayer. (a) Mass density profiles for a DOPC:12chlf bilayer. The profile for ctcl from a DOPC:12ctcl simulation has been also plotted. (b) PMF profile for chlf and ctcl molecules inside the bilayer (bottom) and the trajectory of a chlf molecule tracked for 24 ns (vertical arrow). (c) Profile of the average dipole orientation angle α. In the inset, the preferred orientation of a chlf molecule in the inner lipid/water interface is shown. Green and cyan sticks correspond to DOPC and water molecules, respectively. Green beads represent phosphate groups, whereas chlf has been augmented for clarity: blue beads correspond to Cl atoms and the red bead stands for the H atom.

To assess the molecular origin of anesthesia, membrane properties that show a different response to chlf and ctcl have to be analyzed. Most general anesthetics are known to have permanent dipole moment, contrarily to their non-anesthetic pairs, and NMR experiments have revealed that this difference causes a different partitioning of these compounds inside the bilayer [36]. This differential behavior is captured here for the chlf/ctcl pair, and the results are summarized in Figure 2 for a DOPC bilayer with a clinically relevant chlf concentration (12chlf). First, mass density profiles in Figure 2A show that chlf spans the hydrophobic region of the membrane with a preference for the inner region of the lipid/water interface, close to the phosphate groups. Instead, ctcl is located in most inner region of the membrane with no preference for the lipid/water interface.

Second, inspection of chlf trajectories inside the bilayer reveals that chlf molecules display a very dynamic behavior that combines short confinement periods close to the interface with fast jumps from one leaflet to the other (see Figure 2B). In average, a chlf molecule crosses the interleaflet plane about 5 times per nanosecond, and the mean confinement time (periods where the molecule is closer to the phosphate group than to the interleaflet plane) is about 0.4 ns. This behavior can be explained by means of the variation of the free energy as a function of the position of a molecule along the bilayer normal, the potential of mean force. In Figure 2B, the PMF profiles for chlf and ctcl are plotted. A rather flat shape is observed for ctcl, whereas in the case of chlf two minima close to the lipid/water interfaces are obtained. However, and according to the observed fast interleaflet exchange dynamics, the energy barrier required to jump between the leaflets of the bilayer is very small, of the order of 0.4 k_B_T.

Third, the dipole of chlf molecules shows a preferential orientation when being close to the polar membrane region. The average orientation of the vector connecting the C and H atoms of chloroform (dipole vector) has been calculated. In Figure 2C, the profile of the angle of this vector with the positive z-axis, α, is represented, and a preferential orientation is observed close to both lipid/water interfaces. A closer inspection reveals that chloroform molecules align the H atom to the lipid polar groups and place Cl atoms in the more hydrophobic region. As expected, ctcl does not display any preferential orientation. Finally, DSPC/Chol and/or other chlf fractions show the same behavior than in Figure 2.

**Figure 3:**
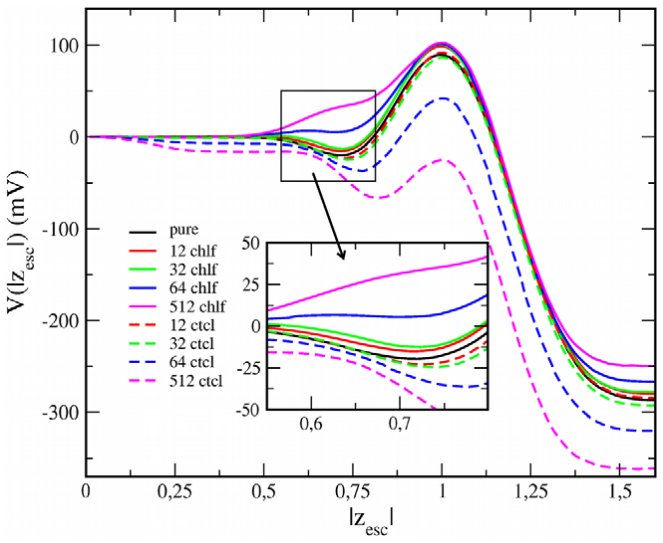
Electric potential profiles for DOPC bilayers with different amounts of chlf and ctcl molecules. The profiles are plotted in the scaled distance to the bilayer center.

The observed particular partitioning and orientation influence the charge distribution inside the membrane, and therefore its electrostatic potential profile. Figure 3 shows the basic characteristics of V(z) for a pure DOPC bilayer. The potential remains flat in the inner hydrophobic region, and shows a minimum when approaching the inner water/membrane interface due to the few water molecules that reach that part of the membrane. Following the outward direction, lipid headgroup dipoles dominate in the interfacial region and strongly increase V(z). In the outer interface section, water dipoles predominate and the potential sharply decays to negative values (water molecules are aligned with their dipoles pointing to the membrane). Addition of chlf mainly affects the inner lipid/water interface: there, chlf dipoles point the opposite direction than water molecules, so that the minimum of V(z) is attenuated and even suppressed at large chlf fractions (Figure 3). Additionally, the total monolayer potential drop is decreased. Instead, ctcl shows a global reduction of V(z) in the interfacial region, mainly due to the indirect effect on the headgroup dipoles, and therefore an increase of the total monolayer drop is observed (Figure 3). Similar effects of chlf and ctcl are found for DSPC/Chol bilayers (data not shown).

**Figure 4:**
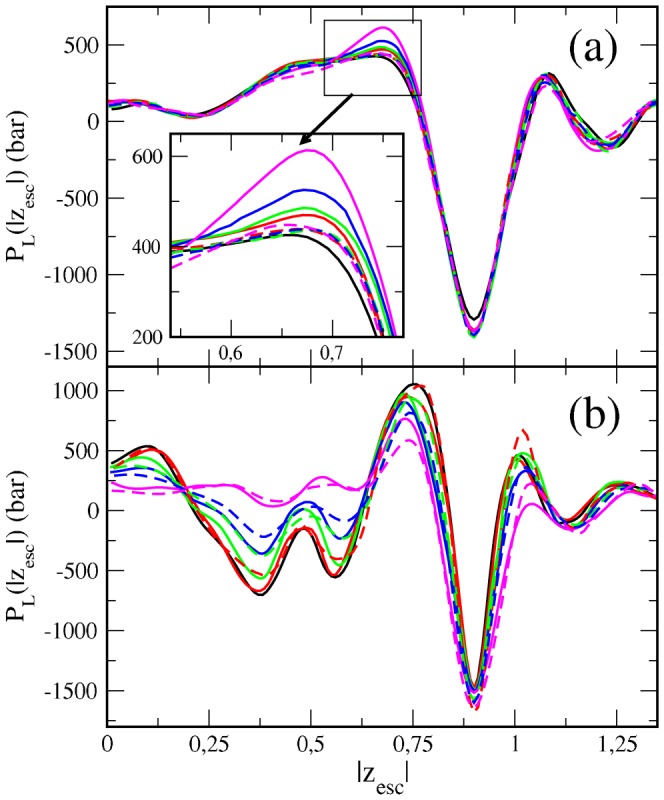
Lateral pressure profiles for (a) DOPC and (b) DSPC/Chol bilayers with different amounts of chlf and ctcl molecules. The profiles are plotted in the scaled distance to the bilayer center. The color code is the same used in Figure 3.

The membrane lateral pressure profiles are also affected by the inclusion of chlf or ctcl. Typical pressure profiles (see black curves in Figures 4A and 4B) display a deep negative pressure peak localized in the interfacial regions as a consequence of the large surface tension between water and lipid hydrocarbon tails. In the inner lipid/water interface sections, the strong repulsion among the rather ordered hydrocarbon segments close to the headgroup result in a positive pressure peak [15], [16]. Figure 4A shows how chloroform increases the positive pressure peak in DOPC (*ld*) membranes, whereas ctcl has a weaker and uncertain effect. Although the influence of chlf at clinically relevant concentrations is relatively small, it is large in magnitude since local pressure values are huge. For example, addition of only 12 chlf molecules increases in 60 bars the lateral pressure of the positive pressure peak at the inner interfacial region.

Due to its condensing abilities, cholesterol increases the magnitude of the positive and negative pressure peaks mentioned above, and more importantly, it causes strong undulations in the hydrophobic part of P_L_(z) (Figure 4B) [37], [38]. Interestingly, as it is observed in Figure 4B, the effect on DSPC/Chol (*lo*) membranes is similar for both chlf and ctcl: the P_L_(z) profiles for the hydrocarbon region of the membrane are progressively smoothed when increasing the organohalogen concentration. The strong oscillations in the profiles do not allow discerning differential effects of chlf and ctcl on the main pressure positive peak as observed for DOPC (*ld*) bilayers.

The reported changes in P_L_(z) profiles caused by chloroform are consistent with the corresponding alterations on the structural membrane properties. Membranes in a *lo* phase are disordered by chlf [5], [6], so that pressure profiles become smoothed. Instead, lipids in *ld* bilayers become ordered preferentially in those tail carbon segments close to the interface where chlf accumulates, thus increasing the positive P_L_(z) peak.

Unfortunately, lateral pressures can not be measured experimentally. Instead, the integrated moments of the profiles correspond to elastic properties of the membrane that can be obtained in the laboratory. For example, the first moment of the lateral pressure profile evaluated along the thickness of each monolayer leaflet, 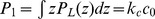, where k_c_ is the elastic bending modulus of each monolayer and c_0_ its spontaneous curvature. For a symmetric bilayer both monolayers display the same spontaneous curvature with opposite sign. As a consequence, the bilayer remains statistically flat and the internal lateral pressure effects are then equivalent to the elastic response of the bilayer to the frustrated spontaneous curvatures of the two monolayer leaflets. The second moment, 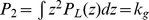, where k_g_ is the Gaussian curvature modulus.

What is important is that the effect of the lateral pressure profiles on the conformational equilibrium of inserted proteins can be characterized in terms of the integral moments of P_L_(z) [39], [40]. In short, the mechanical work needed to change the conformation of a protein between two states can be expressed as 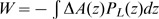, where ΔA(z) is the profile of the difference in cross-sectional area of the protein between the two states. Assuming a Taylor expansion of the area difference profile (namely, a smooth variation across the bilayer) and ignoring higher order terms, the conformational change needs a work proportional to the first and second integral moments of the lateral pressure profiles, 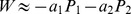 where a_1_ and a_2_ are the expansion coefficients (see [39] and [40] for a detailed derivation). The equilibrium constant of the protein conformational equilibrium can be written as a function of W following standard thermodynamics [39], [40].

The values of P_1_ and P_2_ have been computed for the simulated homogeneous bilayers for the cases with and without organohalogen compounds. The integration limit is taken at the point where the phosphate groups are located (|z_esc_| = 1). Other reasonable integration limits have been tried with similar results. The values are provided in Table 1. For example, for a pure DOPC, P_1_ = −6.2±1 k_B_T/nm and P_2_ = −15.4±2 k_B_T, that are of the order but larger than the estimations for the statistical lattice model proposed by R.Cantor, P_1_ = −15.7 k_B_T/nm and P_2_ = −27.9 k_B_T [39]. The differences may arise due to the absence of headgroup interactions in the lattice model. Atomistic MD simulations reviewed in [41] report a closer value for the first moment in DOPC bilayers; P_1_ = −16.1·10^−12^ J/m (−3.76 k_B_T/nm). Bending modulus and spontaneous curvature have been measured experimentally for DOPE membranes [42] (k_c_ = 0.94·10^−19^ J, c_0_ = −0.351 nm^−1^) leading to k_c_c_0_ = −0.329·10^−19^ J/nm (−7.71 k_B_T/nm), in good agreement with the value of P_1_ obtained here for DOPC. Finally, a coarse-grained molecular model has been recently used to calculate the values for Gaussian curvature in DOPC and DOPE membranes, providing a range from −10 k_B_T to −16 k_B_T [43] depending on the simulation conditions and membrane composition, and in any case, in agreement with the results presented here.

Addition of chlf to DOPC membranes increases (less negative values) the integral moments whereas addition of ctcl has the opposite effect (Table 1). It is interesting to notice here that the lattice model proposed by R.Cantor predicts an increment in the values of the two integral moments when a short-chain alcohol is added to a single-lipid membrane [39], in agreement with what it is observed here for chlf. Instead, addition of both organohalogen compounds to DSPC/Chol bilayers causes a decrease (more negative value) in P_1_ and P_2_ (Table 1).

### Effects on Heterogeneous Model Membranes

**Figure 5:**
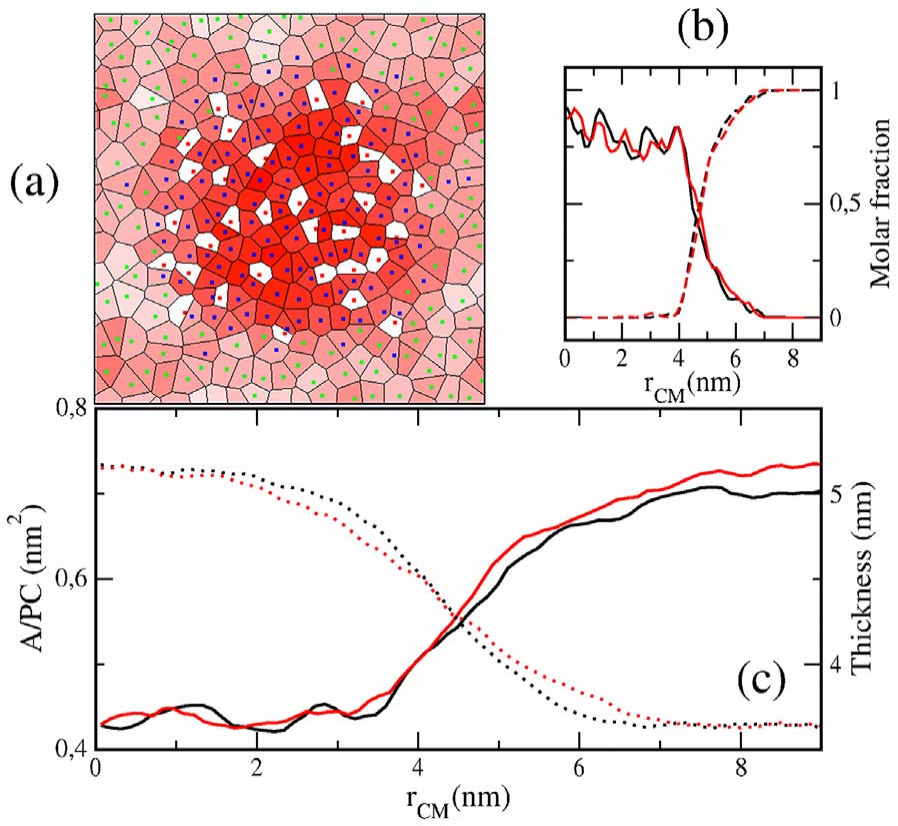
Simulation of the lo patch membrane. (a) Voronoi tessellation of the *lo* patch membrane at the end of the simulation. Polygons are filled with a red color scale proportional to the local membrane thickness. (b) Molar fraction of DSPC (solid) and DOPC (dashed) respect the distance to the center of mass of the nanodomain. (c) Profiles for the area per PC (solid) and thickness (dotted). An average over the last 48 ns of simulation of the chlf-free (black) and chlf-containing (red) *lo* patch bilayers are plotted in panels (b,c).

The simulations of structured lipid membranes show some general properties that are summarized in Figure 5 for the case of the *lo* nanodomain. After 200 ns, the domain remains immiscible and the bilayer system displays a heterogeneous distribution of its structural properties (Figure 5A). Some of these properties have been averaged for the last 48 ns of simulation and plotted respect to the distance to the center of mass of the DSPC/Chol patch, r_CM_. In Figure 5B, the composition profiles show a rather sharp interface (≈2 nm thick) indicating *lo*/*ld* immiscibility. Area per PC and membrane thickness profiles are presented in Figure 5C, and a similar behavior is found for the chain ordering parameter (data not shown). The values at the edges of the profiles are in agreement with the ones reported in Table 1 for the homogeneous *lo* and *ld* membranes. These values are connected by a gradual variation that crosses the domain boundary and spans about 4 nm. DSPC molecules at the edge of the domain (3 nm<r_CM_<5 nm) show a larger area, smaller thickness and higher chain disordering than those typical of a homogeneous DSPC/Chol membrane. In turn, DOPC molecules close to the domain boundary (5 nm<r_CM_<7 nm) are influenced by the ordered phase and display a smaller area, larger thickness and higher chain ordering than those far from the domain.

Addition of 128 chlf molecules does not modify the general behavior explained above (red curves in Figures 5B and 5C). The mean chlf fraction is the same than in the homogeneous bilayers with 32 chlf, so that no significant changes are expected for the membrane thickness at both phases (see Table 1). Instead, the values of area per PC should increase in a 4–5% at the edges of the profile, but this is only observed for the *ld* phase as it will be explained later (Figure 5C). Composition profiles are not altered (Figure 5B), so that *lo*/*ld* immiscibility seems to be preserved upon addition of chlf in the simulated time scales. In order to analyze the stability of the *lo*/*ld* coexistence, additional properties have been monitored: the distribution of neighbor molecules for each PC species, the number of molecules forming the ordered patch, and the correlation of area per PC and chain ordering between transbilayer neighbors. All these properties show that inclusion of chlf molecules does not modify the stability of the nanodomain. Notice, however, that considering a typical lipid diffusivity of 1 µm^2^/s, the mean molecular displacement after 200 ns is of the order of half nanometer; so little information about phase stability can be expected. Longer simulations, only available with coarse-grained MD, sampling a few microseconds would be required to be conclusive about this respect.

### Accumulation in Disordered Lipid Phases

**Figure 6:**
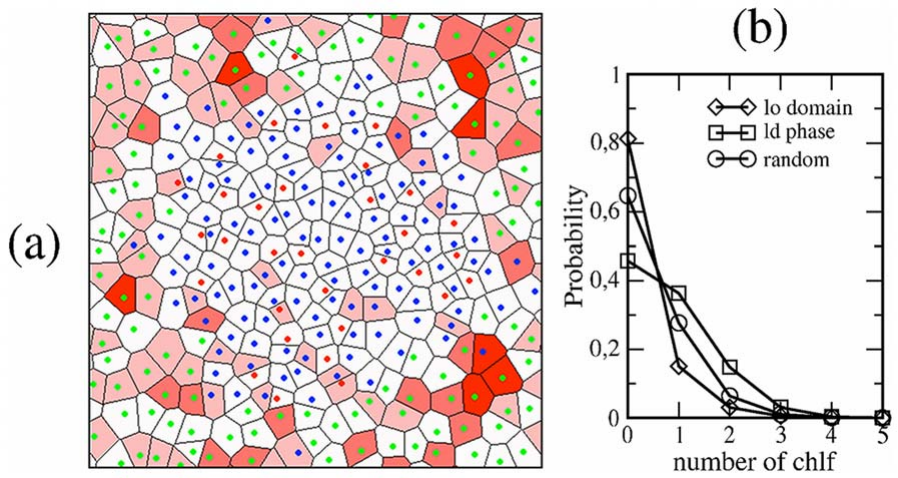
Simulation of the *lo* patch membrane with chlf. (a) Voronoi tessellation of the *lo* domain chlf-containing membrane at the end of the simulation. Polygons are filled with a red color scale proportional to number of contained chlf molecules. (b) Probability distribution of finding a number of chlf molecules close to a molecule in the *lo* domain (DSPC, Chol) or in the *ld* phase (DOPC). The random distribution is provided.

The most relevant observation corresponds to the behavior of chloroform in the bilayer plane. Lateral diffusion of chlf is much faster than for lipid molecules: chlf diffusivity is ≈30 µm^2^/s when placed in the *lo* domain and ≈600 µm^2^/s in the *ld* phase. Even in the slowest phase, the mean displacement of chlf is about 3–4 nm after 200 ns, so chloroform molecules are able to explore the different phases present in the simulated system. In Figure 6, it is observed that chloroform displays a clear preference to be located in the disordered phase: visual inspection of Figure 6A reveals that the *lo* domain has lost most of their contained chlf molecules in favor of the surrounding *ld* phase. The statistics of the last 48 ns of simulation shows that most of the DSPC and Chol molecules (>80%) do not have any chlf at their surroundings, whereas the fraction of DOPC molecules with one or more proximal chlf molecules is much larger than for the molecules in the nanodomain and also than the corresponding for a random distribution (Figure 6B). Although the nanodomain covers the 47% of the area of the bilayer, it only contains the 25% of all chlf molecules.

**Figure 7:**
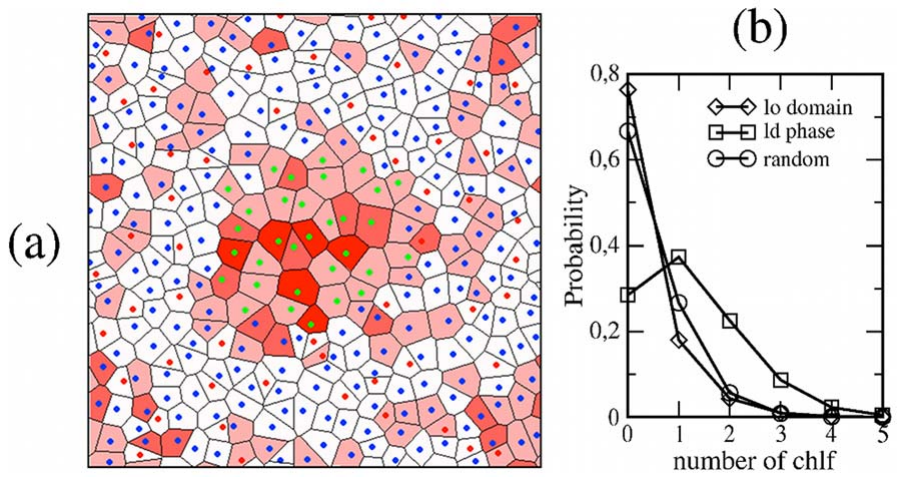
Simulation of the ld patch membrane with chlf. (a) Voronoi tessellation of the *ld* domain chlf-containing membrane at the end of the simulation. Polygons are filled with a red color scale proportional to number of contained chlf molecules. (b) Probability distribution of finding a number of chlf molecules close to a molecule in the *ld* domain (DOPC) or in the *lo* phase (DSPC, Chol). The random distribution is provided.

Chloroform accumulation in disordered phases is even more evidenced in the simulation corresponding to the small *lo* domain, as it can be observed in Figure 7A. Actually, a detailed inspection of this figure reveals that DSPC molecules far from the domain are richer in chlf than those close to the nanodomain, thus indicating that the equilibrium *lo*/*ld* partitioning of chlf has not been achieved yet at the end of the simulation (100 ns). The distribution for the number of proximal chlf molecules is averaged in the last 48 ns of simulation and plotted in Figure 7B. Depletion and accumulation of chlf are observed in the lipid ordered and disordered phases, respectively. Notice, for example, that about a 33% of DOPC molecules have two or more proximal chlf molecules, that corresponds to the saturation limit ratio (2–3 chlf per PC) measured experimentally [5]. The average of chlf∶PC ratio in the last 5 ns of simulation is 0.8 for DOPC, more than three times larger than the initial (random) ratio (0.25), and it is expected to increase by expanding the simulations until the equilibrium chlf partitioning would be reached. It is not the goal of the simulations of structured membranes to determine the exact chlf equilibrium partitioning in *lo* and *ld* coexistent phases. Instead, the results from these simulations, and particularly from the small *ld* nanodomain, are conclusive and demonstrate the large affinity of chlf to concentrate in highly disordered lipid phases, so that a global low (clinical) chloroform fraction can be locally increased up to concentrations that cause significant alterations of membrane transversal properties as reported previously. The combination of both transversal and lateral partitioning of chloroform in the cell membrane could be therefore important to understand its anesthetic action.

## Discussion

I have reported the results from atomistic MD simulations of lipid membranes subjected to the effect of two organohalogen compounds: chloroform that is an anesthetic, and its non-anesthetic counterpart, carbon tetrachloride. The simulations have been performed in homogeneous ordered and disordered bilayers, and also in heterogeneous membranes composed of segregated ordered and disordered regions.

The comparison between chlf and ctcl in homogeneous bilayers reveals that these compounds partition differently inside the bilayers. Actually, the main difference between many anesthetics and their non-anesthetic counterparts is that the former possess a dipole moment. This implies that once inside the lipid bilayer, anesthetics show a preference to be located close to the inner lipid/water interface also displaying a preferential orientation, whereas the non-anesthetic pairs (lacking in electric dipole) accumulate preferentially in the most inner membrane region. This differential behavior seems to be common for many anesthetics, as revealed in MD simulations [15], [16], [19], and results, in turn, in a differential modification of membrane transversal properties that has been characterized here for the chlf/ctcl pair.

In particular, chloroform (contrarily to ctcl) is found to locally increase the electric potential in the inner lipid/water interfacial region in both *lo* and *ld* phases. The electrostatic properties of a membrane are known to be important in the conformation and functionality of ion channel proteins. Many voltage-gated ion channels open and close due to the motion of voltage sensor domains in response to changes in the membrane electric field [44], [45]. For example, Na^+^, K^+^ and Ca^2+^ channels are made up of four S1, S2, S3, S4 voltage-sensing peptide domains. In particular, the N-terminal segment of each S4 domain contains four Arginine residues that are crucial in the voltage-sensing mechanism [44]. The Arginine residues show a preference to interact with the lipid phosphate groups, whereas the remaining residues are hydrophobic. Due to this amphiphilic nature, the N-terminal segment of the voltage-sensing domain S4 is located at the inner lipid/water interface, as it is shown by MD simulations [46]. On the basis of the above discussion, it is appealing to consider the possibility that an alteration of the electric properties in this particular section of the membrane is critical and may modify the rate of transition between closed and open states of protein channels with the subsequent alterations in membrane excitability.

Alterations in lateral pressure profiles have been also reported. Both organohalogen compounds have been found to disorder the lipids tails in *lo* chol-containing membrane phases, so that pressure profiles in the hydrophobic section become smoothed. Instead, lipids in *ld* bilayers become ordered preferentially in those tail carbon segments close to the interface where chlf accumulates, thus increasing the positive P_L_(z) peak. Interestingly, this latter observation is exclusive of chlf. This differential effect in *ld* bilayers is also evidenced by the response in the variation of the lateral pressure integral moments, P_1_ and P_2_. Addition of chlf increases both moments, whereas inclusion of ctcl has the opposite effect. The relevance of the latter observations, exclusive of chlf, in a plausible lipid-mediated mechanism for anesthesia is supported by the fact that the internal distribution of lateral stresses in a membrane is believed to modulate the functionality of inserted proteins [47]. Using statistical thermodynamics arguments, R.Cantor suggested that local changes in the lateral pressure profile near the lipid/water interface may affect the activity of channel membrane proteins involved in nerve conduction [15], [16]. More specifically, the incorporation of compounds that accumulate in the lipid/water interface increases the local lateral pressure in that region, and therefore, the channel opening will require greater work so that the protein conformational equilibrium will be shifted to favor the closed state [15], [16]. Results reported in [19] provide numerical and experimental evidences that support this hypothesis for lipid membranes containing ketamine. In this case, however, ketamine acts on the lipid/water interface by reducing the local lateral pressure and therefore decreasing the lateral pressure moments.

The reported differential effects of chlf and ctcl on the electric and lateral pressure properties of the membrane suggest plausible indirect lipid-mediated mechanisms to understand the anesthetic action of chlf. It could be argued, however, that the reported effects seem to be *relatively* weak at pharmacological chloroform fractions. For example, addition of 12 chlf molecules causes an increase of about 5 mV and 60 bars of electrostatic potentials and lateral pressure profiles in the inner lipid/water interface, respectively. These quantities are relatively small respect to the absolute values of both transversal properties. For example, although the increase of the lateral pressure is large in absolute values, it only represents a 15% in relative terms (≈400 bar to ≈460 bar). The question is, therefore, how large these changes should be in order to imply significant variations in protein channel functions and induce anesthesia?. Some estimations reveal that changes of only 10 mV may modify by a factor of 150 the opening probability of a voltage-sensing channel protein [45], whereas an increase of a 10% of the lateral pressure at the membrane interface may result in an overwhelming shift in the conformational population of mechanosensitive channel proteins [15], [16]. MD simulations of lipid membranes containing a channel protein (at least, a part of it) could help to determine the required magnitude of the alterations in electrostatic potential and/or lateral pressure profiles to significantly modulate channel proteins function. At this point it is also important to be critical with the method used in this Paper to compute lateral pressure profiles in order to assess the applicability of the obtained results in biological scenarios. The computation of the lateral profiles is performed on very simple membranes formed by a single lipid phase and considering a flat configuration. Under these conditions, the lateral pressure field is decomposed into its planar and perpendicular contributions, the former being assigned to the lateral pressure profile P_L_(z). Instead, the work by Ollila et al. [20] provides new insights on the calculation of the internal pressure field by using a 3D technique that allows the study of more realistic contexts; for example, curved bilayers, membranes composed of different lipid phases and bilayers containing integral proteins. All these scenarios are probably important for the anesthetic action, so the contribution in [20] should be kept in mind in future studies of this issue.

Simulations of structured membranes show that chlf has a strong tendency to accumulate preferentially in those membrane lipid phases that are more disordered. Actually, *ld* phases are those that display clear chlf-induced alterations in both electrostatic and pressure properties. Localization of ion channels to ordered or disordered lipid phases appears to vary depending upon the specific channel [48]. Recently, experimental reconstitution of a voltage-gated K^+^ channel of the Kv family in giant vesicles that display *lo*/*ld* coexistence shows a clear enrichment of the channel in the disordered phase [49]. Therefore, it could be conceived that chloroform may locally reach high enough concentrations in specific highly disordered regions of the membrane and modulate the proteins embedded in those regions by means of the indirect mechanisms outlined above.

The mechanism or combination of mechanisms proposed here does not exclude any other; namely, other mechanisms may act independently of or in conjunction with the ones presented here. Moreover, MD limitations are clear and restrict the conclusions to processes/mechanisms developing at very short time and length scales. Other mechanisms may act at longer times and/or larger lengths scales, and different numerical techniques should therefore be used for their investigation. For example, the high affinity of chlf for *ld* lipid phases indicates that this compound may alter the thermodynamics of *lo*/*ld* (liquid-liquid) coexistence in lipid membranes. This idea is particularly attractive in the context of the lipid raft hypothesis [50], which proposes the formation of *lo* nanodomains (rafts) as a fundamental organizing principle of the components of the cell membrane (lipids and proteins). Chloroform may alter the thermodynamics of *lo*/*ld* coexistence, providing a mechanism for anesthesia at a global scale involving lipid-mediated protein reorganization in the cell membrane. Large-scale coarse-grained simulations of multicomponent bilayers should be used to numerically explore this possibility. Additionally, experiments on giant unilamellar vesicles as those used in [49] can be specifically designed to explore the results presented in this paper.
